# Expert consensus on management of instrument separation in root canal therapy

**DOI:** 10.1038/s41368-025-00372-w

**Published:** 2025-06-09

**Authors:** Yi Fan, Yuan Gao, Xiangzhu Wang, Bing Fan, Zhi Chen, Qing Yu, Ming Xue, Xiaoyan Wang, Zhengwei Huang, Deqin Yang, Zhengmei Lin, Yihuai Pan, Jin Zhao, Jinhua Yu, Zhuo Chen, Sijing Xie, He Yuan, Kehua Que, Shuang Pan, Xiaojing Huang, Jun Luo, Xiuping Meng, Jin Zhang, Yi Du, Lei Zhang, Hong Li, Wenxia Chen, Jiayuan Wu, Xin Xu, Jing Zou, Jiyao Li, Dingming Huang, Lei Cheng, Tiemei Wang, Benxiang Hou, Xuedong Zhou

**Affiliations:** 1 State Key Laboratory of Oral Diseases & National Center for Stomatology & National Clinical Research Center for Oral Diseases & Department of Cariology and Endodontics, West China Hospital of Stomatology, Sichuan University, Chengdu, China; 2 Department of Endodontics, Xiangya Stomatological Hospital, Central South University, Changsha, China; 3 Department of Cariology and Endodontics, Wuhan University & State Key Laboratory of Oral & Maxillofacial Reconstruction and Regeneration, Key Laboratory of Oral Biomedicine Ministry of Education, Hubei Key Laboratory of Stomatology, School & Hospital of Stomatology, Wuhan University, Wuhan, China; 4 State Key Laboratory of Oral & Maxillofacial Reconstruction and Regeneration, National Clinical Research Center for Oral Diseases, Shaanxi Key Laboratory of Oral Diseases, Department of Operative Dentistry & Endodontics, School of Stomatology, The Fourth Military Medical University, Xi’an, China; 5 Department of Conservative Dentistry and Endodontics, School and Hospital of Stomatology, China Medical University, Liaoning Provincial Key Laboratory of Oral Diseases, Shenyang, China; 6 Department of Cariology and Endodontology, Peking University School and Hospital of Stomatology, National Center for Stomatology & National Clinical Research Center for Oral Diseases & National Engineering Research Center of Oral Biomaterials and Digital Medical Devices & Beijing Key Laboratory of Digital Stomatology & NHC Key Laboratory of Digital Stomatology & NMPA Key Laboratory for Dental Materials, Beijing, China; 7 Department of Endodontics, Shanghai Ninth People’s Hospital, Shanghai Jiao Tong University School of Medicine; College of Stomatology, National Clinical Research Center for Oral Diseases, National Center for Stomatology; Shanghai Key Laboratory of Stomatology, Shanghai, China; 8 Department of Conservative Dentistry and Endodontics, Shanghai Stomatological Hospital & School of Stomatology, Shanghai Key Laboratory of Craniomaxillofacial Development and Diseases, Fudan University, Shanghai, China; 9 Department of Operative Dentistry and Endodontics, Hospital of Stomatology, Guanghua School of Stomatology, Sun Yat-Sen University & Guangdong Provincial Key Laboratory of Stomatology, Guangzhou, China; 10 Department of Endodontics, School and Hospital of Stomatology, Wenzhou Medical University, Wenzhou, China; 11 Department of Endodontics, The First Affiliated Hospital of Xinjiang Medical University, College of Stomatology of Xinjiang Medical University, Urumqi, China; 12 Institute of Stomatology, Nanjing Medical University & Department of Endodontics, Affiliated Hospital of Stomatology, Nanjing Medical University, Nanjing, China; 13 Stomatology Hospital, School of Stomatology, Zhejiang University School of Medicine, Hangzhou, China; 14 Nanjing Stomatological Hospital, Affiliated Hospital of Medical School, Nanjing University, Nanjing, China; 15 State Key Laboratory of Oral Diseases & National Center for Stomatology & National Clinical Research Center for Oral Diseases & West China Hospital of Stomatology, Sichuan University, Chengdu, China; 16 Department of Endodontics, School and Hospital of Stomatology, Tianjin Medical University, Tianjin, China; 17 Department of Endodontics, The First Affiliated Hospital of Harbin Medical University & Department of Endodontics, School of Stomatology, Harbin Medical University, Harbin, China; 18 Fujian Key Laboratory of Oral Disease & Fujian Provincial Engineering Research Center of Oral Biomaterial & Stomatological Key Lab of Fujian College and University, School and Hospital of Stomatology, Fujian Medical University, Fuzhou, China; 19 Stomatological Hospital of Chongqing Medical University, Chongqing Key Laboratory of Oral Diseases and Biomedical Sciences, Chongqing Municipal Key Laboratory of Oral Biomedical Engineering of Higher Education, Chongqing, China; 20 Department of Endodontics, School and Hospital of Stomatology, Jilin University, Changchun, China; 21 Department of Endodontics, School and Hospital of Stomatology, Cheeloo College of Medicine, Shandong University & Shandong Key Laboratory of Oral Tissue Regeneration & Shandong Engineering Laboratory for Dental Materials and Oral Tissue Regeneration & Shandong Provincial Clinical Research Center for Oral Diseases, Jinan, China; 22 Jinan Stomatological Hospital, Jinan, China; 23 Hohhot Stomatology Hospital, Inner Mongolia, China; 24 Department of Endodontics, Beijing Stomatological Hospital, School of Stomatology, Capital Medical University, Beijing, China; 25 College & Hospital of Stomatology, Guangxi Medical University, Nanning, China; 26 Department of Endodontics, Affiliated Stomatological Hospital of Zunyi Medical University, Zunyi, China; 27 State Key Laboratory of Oral Diseases & National Center for Stomatology & National Clinical Research Center for Oral Diseases & Department of Pediatric Dentistry, West China Hospital of Stomatology, Sichuan University, Chengdu, China; 28 Department of Oral Radiology, Nanjing Stomatological Hospital, Affiliated Hospital of Medical School, Institute of Stomatology, Nanjing University, Nanjing, China

**Keywords:** Dental diseases, Endodontics, Dental treatment planning

## Abstract

Instrument separation is a critical complication during root canal therapy, impacting treatment success and long-term tooth preservation. The etiology of instrument separation is multifactorial, involving the intricate anatomy of the root canal system, instrument-related factors, and instrumentation techniques. Instrument separation can hinder thorough cleaning, shaping, and obturation of the root canal, posing challenges to successful treatment outcomes. Although retrieval of separated instrument is often feasible, it carries risks including perforation, excessive removal of tooth structure and root fractures. Effective management of separated instruments requires a comprehensive understanding of the contributing factors, meticulous preoperative assessment, and precise evaluation of the retrieval difficulty. The application of appropriate retrieval techniques is essential to minimize complications and optimize clinical outcomes. The current manuscript provides a framework for understanding the causes, risk factors, and clinical management principles of instrument separation. By integrating effective strategies, endodontists can enhance decision-making, improve endodontic treatment success and ensure the preservation of natural dentition.

## Introduction

Instrument separation refers to the fracture of endodontic instruments during root canal therapy (RCT). The etiology of instrument separation is multifactorial, influenced by factors such as the intricate complexity of root canal anatomy, the mechanical properties of endodontic instruments, the techniques employed during instrumentation, and the sterilization procedures. These factors can contribute to instrument fracture within the root canal due to cyclic fatigue, flexural resistance and torsional failure.^1^ Instrument separation is one of the most common complications encountered during RCT, with reported separation rates ranging from 0.25% to 10.0%.^2,3^ Specifically, the incidence of fractured stainless steel instruments varies between 0.25% and 6%, while for nickel-titanium (NiTi) instruments, the rate ranges from 1.3% to 10%.^2–7^ During RCT, instrument separation can hinder the complete cleaning, shaping, and obturation of the root canal. If the separated instrument (SI) can be retrieved, the treatment may proceed as planned. However, severe complications may arise during the retrieval process, such as perforations, substantial loss of tooth tissues, root fractures, and in severe cases, even tooth extraction. Instrument fracture not only disrupts the treatment process but also leads to frustration for both patients and clinicians. Patients may be concerned about the potential health implications of a fractured instrument remaining in situ, especially if they are unaware of its possible consequences.^8^ For endodontists, instrument separation often represents an unsatisfactory outcome, leading to patient complaints or even medico-legal issues.^9^ Therefore, the decision to retrieve SI and the methods employed for retrieval remain significant clinical challenges. The current manuscript will provide a comprehensive framework for understanding the etiology, risk factors, and clinical management of instrument separation in RCT, guiding endodontists in effective decision-making and retrieval strategies to optimize treatment success and preserve natural dentition.

## Factors affecting endodontic instrument separation

**Fig. 1:**
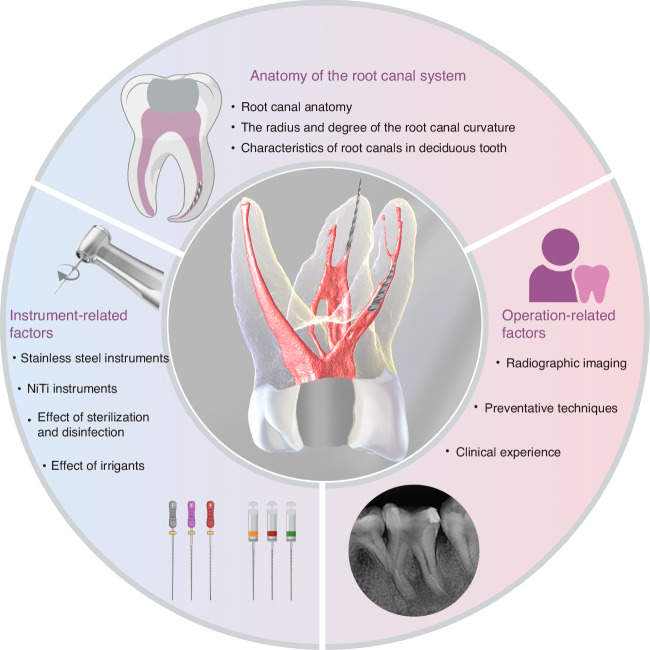
Factors related to instrument separation

The main causes of endodontic instrument separation include the anatomy of the root canal system, instruments-related factors, and instrumentation techniques (Fig. 1).

### Anatomy of the root canal system

The anatomical diversity and complexity of the root canal system are critical factors contributing to instrument separation.

**Fig. 2:**
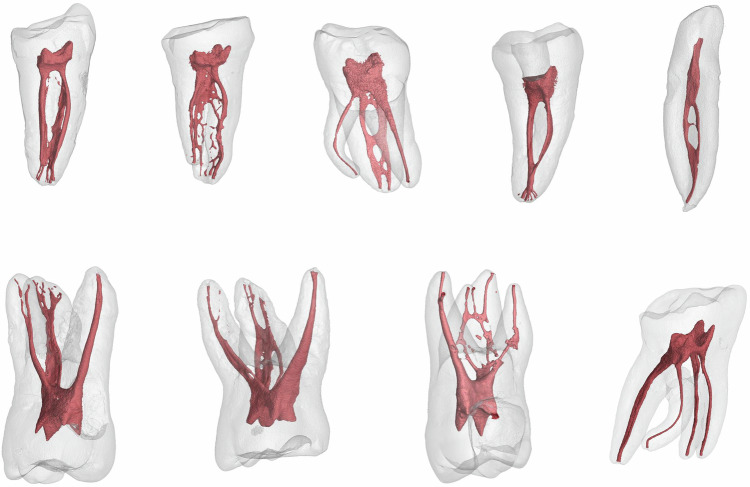
Complex root canals anatomy increases the risk of instrument separation
- **The radius and degree of the root canal curvature** The curvature of a root canal is defined by its angle and radius, both of which significantly affect the fatigue resistance of endodontic instruments. NiTi instruments are more likely to fracture when the root canal curvature exceeds 30°, typically occurring in the middle or apical portions of the curved canals.^18,19^ The radius represents the canal’s abruptness, with smaller radii indicating higher curvatures. As the radius decreases, the risk of fracture increases due to the higher stress and torsional forces on the instrument.^20,21^
- **Characteristics of root canals in deciduous tooth** Root canals in deciduous teeth, particularly in primary molars, often exhibit significant curvatures. These curvatures complicate the use of endodontic instruments, leading to uneven stress distribution at the bends, which increases the risk of instrument separation. Root canals may become narrow and calcified as children age, which increases the resistance to endodontic instruments and potentially resulting in lodged or separated instruments.^22,23^ Understanding these anatomical features of deciduous root canals allows endodontists to adopt preventive measures, reducing the risk of instrument separation and improving the success rate of RCT.
- **Age-related alterations in the root canal system** With aging, dentin continuously deposits along the inner walls of the root canal, gradually narrowing or even completely calcifying and obstructing the canals. This process complicates the use of endodontic instruments, making them more likely to become lodged or experience uneven stress, thereby increasing the risk of instrument separation.^24^

- **Root canal anatomy** Molars, particularly the mesiobuccal canal of the maxillary first molar and the mesial canal of the mandibular first molar, often exhibit complex morphology such as curvatures and the existence of multiple canals within each tooth, thereby increasing the risk of instrument separation.^3,10^ Studies reveal that instrument separation is more prone to occur in narrow and curved canals, and the failure of rotary NiTi instruments appear more often in molars.^6^ Furthermore, mandibular incisors and premolars frequently exhibit a higher prevalence of two root canals, further complicating the treatment process. In addition, root canals with acute curvature, Type V (1–2) root canals, and irregular shapes with multiple ramifications are also more susceptible to instrument separation.^11–13^ Moreover, the incidence of C-shaped root canals is notably higher among Chinese individuals, especially in mandibular second molars.^14,15^ This intricate anatomical configuration renders cleaning and shaping more arduous, thereby increasing the risk of instrument separation.^16,17^ (Fig. 2).

### Instrument-related factors

Various instruments are employed during RCT, including preparation tools, ultrasonic tips, irrigation needles, spiral fillers, and silver points. These instruments are made from different materials such as stainless steel, NiTi, and carbon steel, each with properties tailored to specific functions in the treatment process.^25,26^ While advancements in material selection and design have significantly improved treatment success rates, the inherent properties of the materials and the complexity of root canal anatomy also pose risks of instrument separation, particularly in molars and curved root canals.^27^

- **Stainless steel instruments** Fracture of stainless steel hand files typically occurs after visible deformation of the instrument. Stainless steel instruments used in RCT can experience material fatigue due to torsional and bending stresses. Due to the high hardness and relatively low ductility of stainless steel, these stresses gradually initiate microcracks that propagate under repeated stress until fracture occurs. Additionally, defects in the quality or manufacturing of stainless steel instruments, such as files and reamers, may contribute to fractures.^11^ Microdefects in the grain structure during production can serve as crack initiation sites in areas of stress concentration. Cold working during manufacturing enhances the hardness of stainless steel but reduces its toughness, making the instruments more prone to brittle fractures under localized overload or high-frequency bending.
- **NiTi instruments** The fracture of NiTi instruments is closely associated with their material properties, manufacturing processes, and the mechanical loads encountered during clinical use.^28^ Most NiTi instruments are manufactured through a milling process rather than twisting, which can introduce surface imperfections such as pits, grooves, cracks, and regions of metal rollover. These defects act as stress concentration points, promoting crack initiation and propagation, which may ultimately lead to instrument fracture.^29–31^ Additionally, oxide particles may be incorporated into the NiTi alloy during the manufacturing process. These particles can serve as nucleation sites for micro-voids under stress, further compromising the material’s strength and durability.^32^ Instrument fatigue is influenced by multiple factors, including instrument design, manufacturing processes, root canal anatomy, clinical technique, and sterilization protocols.^33^ NiTi rotary instruments are particularly susceptible to two main types of fatigue: cyclic and torsional. Cyclic fatigue results from repeated tensile and compressive stresses as the instrument rotates within curved canals, leading to the initiation and propagation of microcracks. Torsional fatigue occurs when the instruments lodge within the canal while continue to rotate, generating torsional stress that can result in fracture. There is no definitive number of times a file can be used before fracture.^34^ Some studies advocate for single use, especially in anatomically complex canals, while others suggest that with careful inspection and depending on canal morphology, reuse up to 3–5 times or more may be feasible.^35,36^ Regular examination of the cutting edges for signs of wear and strain is essential to ensure safe and effective performance. Any signs of deformation, unwinding, dulling of the cutting flutes, or loss of luster should warrant immediate disposal. Endodontists should also maintain detailed records of each instrument’s usage history, including the number of uses and the type of canals treated to better evaluate its condition and risk of fracture.
- **Effect of sterilization and disinfection** Endodontic instruments undergo cleaning and sterilization before their initial use and prior to each reuse. However, the impact of these procedures on instrument fracture remains debate. Research suggests that repeated exposure to sodium hypochlorite and autoclave sterilization may slightly reduce the torsional strength of stainless steel instruments, but the clinical implications seem minimal.^37–39^ For NiTi instruments, multiple sterilization cycles can lead to surface alterations, including corrosion and defect, resulting in increased surface roughness. Despite these alterations, no definitive correlation has been established between surface changes and instrument separation.^40–44^ Sterilization by dry heat and autoclave affects the cyclic fatigue resistance and torsional strength of various NiTi instruments, with some studies reporting enhanced fracture resistance.^45–49^ While sterilization may raise certain concerns, the clinical significance of these findings remains unclear, requiring further investigation to better understand their implications for endodontic practice.
- **Effect of irrigants** The use of irrigants during instrumentation also affects the risk of instrument separation.^50,51^ Prolonged exposure of NiTi and stainless steel instruments to high-concentration sodium hypochlorite, particularly at elevated temperatures, has been shown to induce corrosion.^52^ This corrosion can create microscopic surface defects, thereby reducing the cyclic fatigue resistance of the instruments.^53^ Chelating agents in some irrigants may rapidly deplete the active chlorine in sodium hypochlorite, reducing its cleaning efficacy and potentially worsening the corrosion process.^54,55^ Studies suggest that low-concentration sodium hypochlorite or short-term localized exposure does not significantly impair the cyclic fatigue resistance or torsional strength of NiTi instruments. However, extended exposure or high-temperature treatments can intensify corrosion and fatigue damage, increasing the risk of instrument fracture during clinical use.^53,56,57^ To mitigate the risk of instrument separation, careful management of irrigant concentration, exposure duration, and temperature is crucial.

### Operation-related factors

Radiographic imaging is critical in diagnosing and managing instrument separation during RCT. Preoperative imaging enables endodontists to anticipate potential anatomical challenges, such as root canal curvatures or calcifications, thereby reducing instrument separation risk. Inadequate access cavity preparation, such as incomplete removal of the pulp chamber roof or failure to establish a straight-line access to the root canal, significantly increases the risk of instrument fracture. Additional contributing factors include excessive force, high rotational speed, and skipping file sizes during root canal instrumentation.^58,59^ Repeated use of stainless steel or NiTi files exhibiting visible defects, such as cracks, uneven flute spacing, shiny marks, unwinding, sharp bends, permanent distortions, or signs of corrosion significantly increases the risk of instrument separation. Improper handling or unfamiliarity with instrumentation procedure can also cause instrument fracture. For instance, allowing a barbed broach or spiral filler to enter narrow curved canals may cause instrument fracture when forcibly withdrawn. Preventative techniques, such as the crown-down technique, has been recommended for the vast majority of rotary NiTi instruments in order to reduce friction and minimize the fracture.^60^ Establishing a continuous glide path of at least size #15 prior to the utilization of the main series of rotary NiTi instruments is another crucial step for preventing fractures.^61–63^ In curved canals, utilizing a reciprocating motion during instrumentation prolongs the lifespan of NiTi instruments and enhances their resistance to cyclic fatigue.^64,65^ The use of torque control electric motors, which limit excessive torque and reverses the direction of rotation when the maximum torque is reached, has significantly reduces the risk of fracture, particularly among less experienced clinicians.^66^ Instrument separation rates tend to decline with increased clinical experience.^1^ Studies show that endodontists encounter fewer incidents of instrument separation compared to general practitioners or less trained individuals.^67^ Experienced endodontists are more proficient at identifying fracture risks and employing advanced techniques to manage challenging cases. Nevertheless, even among highly skilled practitioners, the risk of instrument separation cannot be fully eliminated.^68^

## Preoperative assessment for managing instrument separation

When the instrument separation occurs, the endodontist must thoroughly evaluate the prognosis and treatment complexity. This evaluation involves assessing the feasibility of instrument retrieval and anticipating potential complications, such as perforation, extrusion of the fragment beyond the apical foramen, and secondary instrument separation, etc. Moreover, clear and transparent communication with the patient is essential to ensure understanding of the proposed treatment plan, including its risks, benefits, and alternative options, thereby facilitating informed decision-making and managing expectations. Endodontists should inform patients about potential complications and considerations related to treatment duration, cost, and potential psychological impact, prior to obtaining informed consent. A variety of preoperative factors must be assessed before managing the instrument fragment. These factors include:

### Assessment of the tooth

- **Comprehensive assessment of the affected tooth** To evaluate the prognosis and determine the appropriate management of SI, it is essential to assess the tooth’s retention value and restorability. This includes examining the tooth structure, pulp, periapical and periodontal tissues, and the patient’s systemic health. Teeth with root fractures, advanced periodontal diseases, or no potential for restoration are typically recommended for extraction. Additionally, factors like limited mouth opening can hinder surgical or retrieval procedures, particularly in the posterior region.^1^ Assess the anatomy of the root canal and the root dentin, as these factors have an impact on the visibility and accessibility of the SI. Posterior teeth often have more complex root canal systems, including root curvatures and external concavities that may not be visible in clinical or radiographic examinations, increasing the risk of complications.^69,70^
- **Root morphology** It is vital to examine root morphology, including dentin thickness and the depth of the external concavities. Retrieving SI often requires the removal of dentin, and excessive removal can weaken the root structure, thereby increasing the risk of perforation or root fracture.^71^ For teeth featuring thin canal walls or deep root concavities, bypassing the SI or instrumenting and obturating the root up to the fragment might be safer alternatives. The decision to retrieve the SI should balance the potential for success against the structural integrity of the tooth.^72^
- **Root canal anatomy** The length, diameter and curvature of the root canal significantly impact instrument retrieval feasibility. Severe curvatures, particularly those exceeding 25°, significantly increase the likelihood of NiTi instrument separation.^6,73^ Retrieval success rates decrease from 83% to 43% when the curvature exceeds 20°, with smaller radii further lowering success.^74,75^ Curvatures >30° not only require more time but also significantly reduce the chances of successful retrieval. SI in root canals with mild curvatures and radii exceeding 4 mm are accessible and retrievable, whereas greater curvature and smaller radii complicate the retrieval process and increase risk of complications.^74,76^
- **Evaluation of intracanal and periapical infection** The status of intracanal and periapical infection is a key determinant of prognosis. In vital teeth featuring intact root canal systems and lacking microbial contamination, the long-term prognosis is generally favorable, even if SI remains in the canal.^77^ The timing and location of instrument separation are also critical indicators. Separation near the apex following thorough cleaning suggests a better prognosis, whereas early separation during the initial stages of instrumentation in infected canals can obstruct cleaning and compromise treatment outcomes.^78^ The extent of periapical infection is assessed through clinical and radiographic evaluation of periapical periodontitis and radiolucency. These findings reflect the severity of intracanal infection and influence the decision to attempt SI retrieval or adopt alternative strategies.

### Assessment of SI

**Fig. 3:**
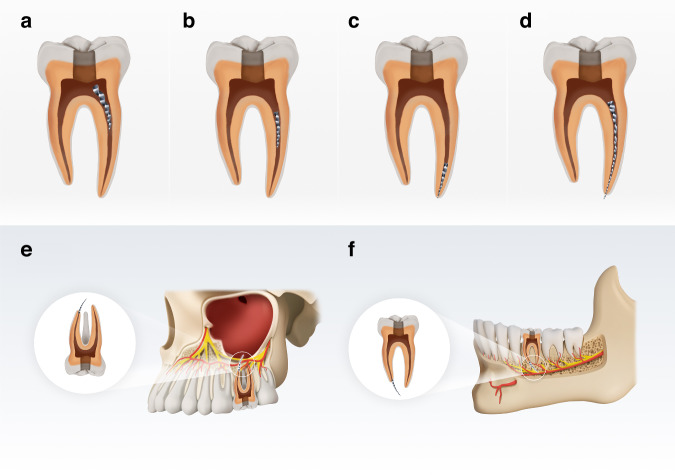
Localization of SI. **a** Instrument protruding into the coronal chamber. **b** Instrument with both ends within the root canal and located at the middle third of the root canal. **c** Instrument with both ends within the root canal and located at the apical third of the root canal. **d** Instrument extending from the pulp chamber into the periapical region (**e**, **f**) Instrument extruded beyond the confines of the tooth in the maxillary sinus (**e**) and (**f**) in the mandibular nerve canal
- **Size of SI** The length and size of the SI significantly impact retrieval success. Longer fragments are generally easier to retrieve due to better accessibility of their coronal portion.^75^ The retrieval time and method vary based on instrument length.^27^ Most separated NiTi instruments have an average length of 3 mm.^6,81^ For each additional millimeter of length, the duration of retrieval attempts increases.^76^ Instrument length also correlates with canal curvature. Longer fragments (>3.1 mm) in highly curved canals (>30°) have a greater contact area with the canal wall, which complicates retrieval and prolongs the retrieval process.^76^ In such cases, ultrasonic techniques alone may be insufficient, necessitating the use of adjunctive techniques, such as micro-tube and wire loop techniques. The instrument’s diameter also influences retrieval complexity. Instruments with large diameters are more prone to separate in curved canals, making retrieval more challenging.^6^ The width of the fracture end indicates the extent of dentin removal needed; larger and longer fragments necessitate more dentin removal, increasing retrieval difficulty and risks.^27^
- **Material of SI** Stainless steel K-files are generally easier to retrieve than NiTi rotary instruments due to differences in taper, cross-sectional design, and mechanics.^11^ NiTi instruments, owing to their rotational motion, are prone to get lodged in canal walls, frequently occluding the entire root canal.^72^ Moreover, the increasing taper of NiTi instruments renders access and trephining around the coronal segment of the instrument more arduous and retrieval more challenging. NiTi instruments are brittle and often disintegrate into fragments when exposed to direct ultrasonic energy during retrieval process due to heat-generated and cyclic fatigue.^82^ The shape-memory properties of NiTi further complicate retrieval, as fragments in curved canals often press against the outer wall rather than staying centered, hindering access and retrieval process.^83^ In contrast, stainless steel instruments are less affected by these factors and are generally easier to retrieve.^84^

- **Localization of SI** The localization of the SI offers fundamental information for decision-making in the management of the instrument. Endodontists should determine whether the instrument is located in the coronal, middle, or apical third of the root canal. Additionally, in curved root canals, it is crucial to determine whether the separation occurred at the upper, lower segments of the curvature.^3,72,79^ The localization relative to the curvature is particularly significant, as most NiTi instrument separations in the apical third of molar canals, often at the curvature^3,6,80^ (Fig. 3).

### Radiographic evaluation

Radiographic evaluation is crucial for diagnosing and managing SI in root canals. Periapical radiographs, though widely used, have limitations due to their two-dimensional nature. They often lack detailed structural information and are susceptible to overlapping anatomical features, which can hinder accurate assessment of surrounding dentin thickness or residual dentin post-retrieval. Cone beam computed tomography (CBCT) offers high-resolution, three-dimensional (3D) imaging of the tooth root and surrounding tissues, offering significant advantages for preoperative evaluation of dentin thickness.^85,86^ In cases of instrument separation, both periapical radiographs and CBCT scans are recommended during the preoperative phase. CBCT is particularly valuable for complex root canal systems, as it enables a comprehensive 3D assessment of the instrument’s location, length, and its spatial relationship with the tooth root. This allows endodontists to evaluate the root canal anatomy, including curvatures, narrow segments, or calcified areas, all of which are factors that increase the complexity and risk of instrument retrieval.^87^ The detailed information helps endodontists anticipate procedural challenges, adjust treatment strategies, and reduce the likelihood of further instrument separation.^88^ While CBCT provides unparalleled imaging for localizing the fragment and assessing the root canal’s 3D structure, it may produce artifacts that hinder the identification of the instrument’s material and type.^89^ In such cases, periapical radiographs can serve as a complementary tool, offering additional information about the instrument’s material properties.^86,90^ Radiographic evaluation also plays an essential role in postoperative follow-up. It can confirm the complete retrieval of the SI and assess the integrity of the RCT. By integrating advanced imaging techniques such as CBCT with traditional radiographs, endodontists can enhance diagnostic accuracy and optimize treatment outcomes.

### Difficulty assessment of retrieval SI

- **SI located in the coronal third of the root canal** Instruments separated in the coronal third of the root canal are generally easier to retrieve, especially if located in straight canals or near the root canal orifice. Their accessibility reduces procedural complexity compared to instruments in curved canals, where retrieval is more challenging.^11,74^
- **SI near the root canal curvature** When SI is near the root canal curvature, retrieval depends on dentin thickness and the ability to create a safe channel from the canal orifice to the instrument’s coronal end. If sufficient dentin thickness is present to create a channel without risking perforation or jeopardizing tooth structure, retrieval may still be possible.^84^
- **SI in the apical region of a curved canal** Retrieving SI from the apical region of a curved canal is considerably more challenging. Establishing a safe retrieval channel is difficult due to anatomical constraints and the risks of excessive dentin removal. Moreover, instrument movement during retrieval attempts may cause the fragment to extrude beyond the apical foramen, increasing risks and reducing the success rate of retrieval. The clinician’s level of expertise plays a critical role in such scenarios. Experienced endodontists are better equipped to navigate the complexities of curved canals, minimize dentin loss, and reduce retrieval time, thereby improving the safety and success of the procedure.^1,68^

## Principles for clinical management of instrument separation

**Fig. 4:**
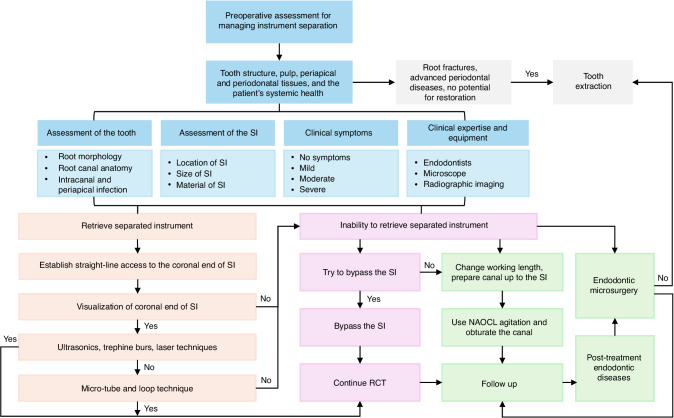
Clinical decision-making strategies in the management of instrument separation

The primary goal in managing instrument separation is to restore the canal’s cleaning and filling pathway, ensuring treatment success. Whenever possible, the SI should be removed. If retrieval is too challenging or risky, alternative approaches include bypassing the instrument or shaping and obturating the canal up to its location. The final decision should account for the tooth’s condition, the instrument’s characteristics, and the clinician’s expertise to maximize treatment outcomes and minimize complications. We have summarized a flowchart for clinical decision-making strategies regarding instrument retrieval (Fig. 4).

### Tooth assessment

The anatomical and structural characteristics of the tooth are critical factors in determining the appropriate management strategy for SI. Factors such as the diameter, length, curvature of the root canal, and dentin thickness greatly influence the treatment approach and procedural complexity.^27,91^ The success rate of retrieving instrument fragments is higher in thick and straight canals. Conversely, narrow or curved canals present increased challenges and risks, necessitating careful evaluation and management.^92^ Additionally, the infection status of the root canal plays a pivotal role. Endodontists must assess whether the canal is infected and the potential risks of treatment failure if the instrument is not retrieved. In non-infected canals or cases with adequate preparation, the absence of retrieval does not necessarily increase the risk of treatment failure.^78^

### Considerations for SI

The instrument’s characteristics, including its diameter, length, and location within the canal, are key considerations in treatment planning.^69^ Shorter or deeper instruments are typically more challenging to retrieve, especially in curved or narrow root canals. Conversely, longer instruments located in straight, wide root canals are easier to access and remove. Techniques such as micro-tube technique can be employed in these cases. If retrieval is deemed beneficial and the associated risks are manageable, it is generally recommended to retrieval the instrument to avoid compromising subsequent canal cleaning and disinfection. If retrieval is not feasible or poses excessive risk, bypassing the instrument or encapsulating it in filling material is a viable alternative to preserve the integrity of the root canal treatment.^93^

### Radiographic diagnosis

Radiographic imaging is essential for diagnosing and managing instrument separation. Preoperative imaging, including periapical radiographs and CBCT, is highly recommended. Periapical radiographs may have limitations, such as image overlap, but they can provide information about the instrument’s material and type. CBCT, on the other hand, offers 3D imaging that enables a more comprehensive assessment of the instrument’s position, length, and relationship to the canal and root walls. CBCT also facilitates preoperative evaluation of dentin thickness, predicting the difficulty of retrieval, and guiding treatment planning. Postoperative radiographic imaging is equally important for confirming instrument removal and assessing the integrity of RCT.^94–96^

### Clinical techniques

The choice of techniques, retrieval, bypassing, or retention should depend on the clinical scenario. Retrieval methods, such as micro-tube and loop technique or ultrasonic method, must be selected with consideration for the canal’s anatomy and the instrument’s characteristics. If retrieval is impractical or too risky, bypassing the instrument or encapsulating it in filling material can preserve the treatment outcome. If neither option is viable, the endodontist should focus on cleaning and shaping the root canal above the SI, ensuring thorough preparation and filling. If treatment fails or symptoms persist, alternative treatments like endodontic microsurgery, intentional replantation, or extraction should be considered.^97,98^ Retrieving the SI also helps alleviate the psychological burden on both the clinicians and the patients, reducing potential medical disputes.^99^

## Techniques for retrieval SI

After thoroughly evaluating the tooth and the SI within the root canal, the first step in retrieving the instrument is to establish straight-line access to its coronal end.^100^

### Establishing straight-line access to the coronal end of SI

**Fig. 5:**
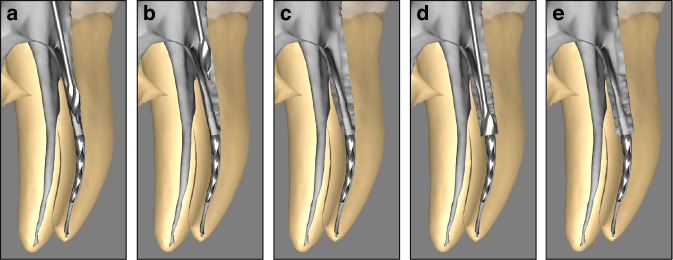
Establishing straight-line access to the coronal end of SI. **a** Approaching the SI using GG drills. **b** Avoiding the furcation while preserving tooth structure. **c** Creating straight-line access to the coronal end of the SI. **d** Preparing a staging platform. **e** Successfully creating a staging platform to provide sufficient lateral space

Establishing straight-line access to the coronal end of SI is a critical first step in various techniques for instrument retrieval.^74,84^ The initial phase involves precisely locating the SI using radiographic examination, magnification with a dental operating microscope, and/or an endodontic endoscope.^101^ This comprehensive evaluation provides detailed information about the instrument’s location, orientation, and depth within the root canal. Once the location is confirmed, a rubber dam should be applied to isolate the operating field, ensuring an aseptic environment and improving visibility. Establishing this access requires meticulous preparation to facilitate the subsequent steps in instrument retrieval. The recommended method involves using hand files to progress from the canal orifice toward the coronal end of the SI. Starting with smaller files and gradually increasing to larger ones helps to enlarge the canal space methodically. This process creates sufficient room for Gates-Glidden (GG) drills or larger NiTi instruments, forming a tapered, straight path to the fragment. To avoid complications such as strip perforation, GG drills or large NiTi instruments should only be used in the root canal’s relatively straight portions. Employing a gentle “brushing” motion directed away from the furcation helps preserve as much tooth structure as possible (Fig. 5a, b). This technique ensures straight-line access to the coronal end of the SI (Fig. 5c). If there is insufficient space around the instrument for the effective use of a fine ultrasonic tip, a staged platform should be prepared (Fig. 5d).^100^ This platform allows for the circumferential removal of dentin around the instrument using the ultrasonic tip. To minimize dentin loss, the endodontist should select an appropriately modified GG drill or NiTi platform drill, based on the dimensions required for the retrieval technique. The size of the ultrasonic tip or trephine bur should correspond to the diameter of the fracture end of the SI, ensuring sufficient lateral space for visualization and access (Fig. 5e). With proper technique and the aid of illumination and microscopy, the coronal end of the SI can be clearly visualized and accessed.

A variety of devices, techniques, and methods are available for retrieving SI from root canals. Successful retrieval often depends on the ability to establish a safe and effective straight-line access to the coronal end of the SI. The most reliable and safe approach involves a combination of tools and techniques, including the use of a dental microscope, ultrasonics, trephine burs, and micro-tube and loops techniques. When employed appropriately, these methods significantly enhance the chances of successful instrument retrieval while minimizing damage to the surrounding tooth structure.

### Ultrasonic technique

**Fig. 6:**
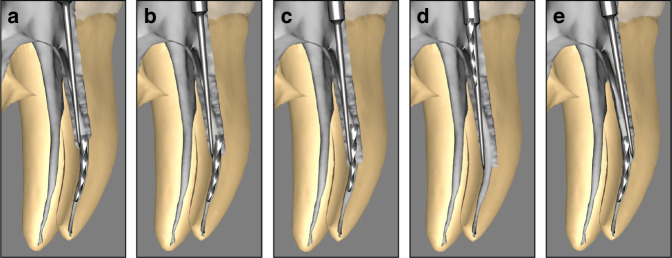
Ultrasonic technique for retrieving SI. **a** Ultrasonic tip removes dentin on one side of the SI. **b** Ultrasonic tip wedges between the SI and the root canal wall. **c** Loosening of the SI. **d** SI “jumps out” of the canal orifice. **e** The ultrasonic tip removes thicker dentin on one side of the SI

When using ultrasonic techniques to remove SI from root canals, it is necessary to operate at a lower power setting to reduce the amplitude of the ultrasonic tip’s movement. After creating straight-line access to the coronal end of the fragment and preparing a staging platform, the ultrasonic tip is initially applied in a semi-circular motion. This motion focuses on one side of the SI’s coronal end, gently removing the surrounding dentin (Fig. 6a). The next step involves carefully wedging the ultrasonic tip between the SI and the root canal wall (Fig. 6b) to loosen the fragment (Fig. 6c), eventually allowing it to “jump out” of the canal orifice (Fig. 6d). If localized ultrasonic movement fails to loosen or dislodge the instrument, the tip should be maneuvered in a counterclockwise circular motion around the instrument. This technique incrementally removes more dentin, exposing the coronal end of the instrument further. Ultrasonic technique often succeeds in loosening the SI, enabling it to rotate out along its long axis. As a classic and effective method, ultrasonic technique offers unique advantages. The ultrasonic tip can be operated under direct microscopic visualization, allowing asymmetrical removal of dentin around the SI. By focusing on only one side of the instrument (Fig. 6e), the method helps preserve dentin on the thinner root canal wall, reducing dentin loss. For optimal visibility, the procedure is typically performed without water irrigation. However, this can increase the risk of heat generation in the periodontal tissues. Prolonged use of ultrasonic tip at elevated temperature may cause secondary separation of NiTi instruments. To mitigate this risk, the ultrasonic tip must be activated at a lower power setting, which may extend the duration of the retrieval procedure and reduce efficiency. This technique demands both advanced theoretical knowledge and considerable practical experience from the practitioner to achieve successful outcomes while minimizing risks.^1,102^

### Trephine bur technique

**Fig. 7:**
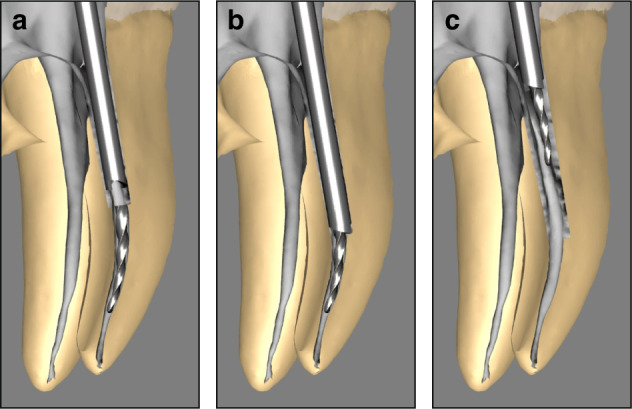
Trephine bur technique for retrieving SI. **a** The trephine bur encircles the instrument and is advanced downward along its long axis. **b** The trephine bur removes dentin around the SI. **c** The SI is carried out within the trephine bur by dentin debris

The trephine bur technique is a specialized method employed during RCT for retrieving fractured instruments. It utilizes a hollow, tube-shaped trephine with a cutting edge at its tip to remove dentin layer by layer around the SI. This approach facilitates precise localization, controlled trephination, and ultimately exposure of the instrument’s coronal end.^103^ Trephine burs are designed with a small, uniform diameter, and their inner diameter is only slightly larger than the SI. This enables conservative removal of the surrounding dentin while minimizing damaging the tooth structural. The hollow, tubular design allows the trephine bur to use the SI as a guide, reducing the risk of slippage, deviation, and lateral perforation. Once straight-line access to the coronal end of the SI and a staging platform are established, an appropriately sized trephine bur is selected based on the instrument’s diameter. The trephine bur is then positioned to encircle the SI and gradually advanced along its long axis (Fig. 7a), steadily removing dentin around it (Fig. 7b). During the procedure, the fragment may become trapped within the trephine bur by dentin debris, allowing for its removal (Fig. 7c). However, if the trephine bur fails to retrieve the instrument after creating a sufficiently deep groove, additional tools such as a micro-tube or loop technique may be required.

### Micro-tube and loop technique

**Fig. 8:**
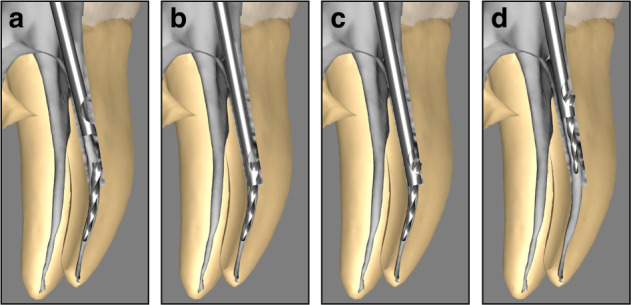
Micro-tube and loop technique for retrieving SI. **a** Inserting the bevel of the micro-tube between the SI and the outer wall of the root canal. **b** Guiding the SI into the micro-tube. **c** Inserting the corresponding wedge into the tube to push the instrument’s head into the side window. **d** Securing and retrieving the SI

The micro-tube and loop technique is an auxiliary method employed to retrieve SI lodged within the root canal. This approach involves either wedging a micro-tube or core pin around the fractured instrument or using loop to secure its end, facilitating the retrieval procedure.^104^ Following an ultrasonic or trephine bur trephination procedure to remove surrounding dentin, NiTi instruments often lodege against the outer wall of the root canal due to their shape memory properties. Even when loosened, the angle between the coronal aspect and the top of the SI may prevent its retrieval. In such cases, the micro-tube or loop technique becomes the most effective or sometimes the only viable method for retrieval.^105^ In curved root canals, the coronal end of the fragment typically rests against the outer wall of the canal. In these situations, the level of the micro-tube can be inserted between the SI and the canal wall to guide the instrument into the tube (Fig. 8a). Once the tube is securely placed (Fig. 8b), a corresponding wedge is inserted into the tube until it contacts the SI. The wedge is then driven in, pushing the head of the instrument into the side window (Fig. 8c). Once the instrument is secured within the tube, it can be carefully rotated and retrieved from the root canal (Fig. 8d).^104,106^

### Laser technique

Laser technology employs photothermal effects to interact with SI or root canal dentin, making it a valuable tool for retrieving SI, especially in complex or narrow root canals. The high precision and minimally invasive nature of lasers can improve treatment success rates and reduce operation time. Erbium-doped yttrium aluminum garnet (Er:YAG) lasers are particularly effective for removing SI in a relatively short time, utilizing various approaches. Melting the dentin surrounding the SI with the laser, thereby assisting in bypassing the fragment with an H-file for retrieval. Directly melting the SI with the laser. Using the laser to melt solder, thereby joining a copper tube to the exposed coronal end of the SI. Welding the SI to a hollow metal tube using the laser.^107^ While laser technique offers promising results, their application in root canals carries potential risks. These include temperature increases in the dentin and periodontal tissues, which may lead to carbonization, melting, or perforation of the root canal wall. Such risks are particularly significant in curved and narrow root canals, necessitating careful operation to ensure patient safety.^108^

## Retrieval of SI in deciduous teeth

When managing SI in deciduous teeth, the decision to retrieval the fragment or extract the tooth should be guided by the location of the SI and the extent of root resorption. Given the thin dentin structure in deciduous teeth, non-invasive or minimally invasive techniques should be prioritized to preserve as much tooth structure as feasible.^109^ If the SI cannot be easily retrieved, leaving it in the canal and proceeding with conventional root canal filling, followed by regular follow-up visits, is generally not recommended. This is because children often fail to adhere to follow-up schedules, and as primary teeth naturally exfoliate, the SI could dislodge into the alveolar bone, potentially interfering with the eruption of permanent teeth. Additionally, there is a risk of the instrument entering the oral cavity during exfoliation, posing a hazard of ingestion or aspiration. In cases where instrument removal proves too challenging or bypassing the instrument is not feasible, tooth extraction becomes a reasonable and safer option. Post-extraction, a collaborative approach involving a pediatric dentist and an orthodontist is essential to assess the need for space maintenance, typically with a space maintainer. Several studies have demonstrated the successful removal of SI from deciduous tooth root canals using microscope-assisted techniques combined with low-power ultrasonic vibration.^22,23^

## Inability to retrieve SI

If SI cannot be retrieved using conventional methods, or if further dentin removal increases the risk of complications, alternative treatments should be considered. These include bypassing the instrument, encapsulating it within filling material, or leaving it in the root canal while cleaning and shaping the canal above it, followed by preparation and filling up to the instrument’s location. The feasibility of bypassing the instrument largely depends on the available space surrounding it within the root canal. A small pre-curved hand file (#8 or #10) can be used to attempt bypassing. The file should be gently guided alongside the instrument with minimal pressure to avoid damage. If successful, progressively larger hand files can be employed to create a path towards the apex. Oval-shaped canals are generally easier for bypassing, while round canals, where the instrument fully obstructs the canal, are more challenging. If bypassing fails and the file cannot pass the instrument, excessive force should be avoided to prevent perforation. Regardless of the chosen approach, the canal should be thoroughly disinfected with a substantial volume of sodium hypochlorite to reduce microbial colonization and mitigate infection risks before filling. Teeth with SI should be closely monitored over the long term to track symptoms and the healing of periapical lesion. If symptoms such as pain or infection persist, additional treatments such as endodontic microsurgery, intentional replantation, or extraction may be required. Careful case selection and tailored treatment strategies are crucial to ensuring successful outcomes for retreatment cases involving instrument separation.^100^ When instruments inadvertently enter adjacent anatomical structures like the maxillary sinus or mandibular nerve canal, patients may experience pain, inflammation, or numbness (Fig. 3). These situations typically necessitate complex interdisciplinary management. Collaboration among endodontists, oral surgeons, and otolaryngologists is essential in such cases. A multidisciplinary team (MDT) consultation, supported by advanced radiographic imaging, is essential for developing a comprehensive treatment plan. For mild symptoms, conservative approaches such as anti-inflammatory medications and localized management may be sufficient. In cases of severe or persistent symptoms, surgical or endoscopic techniques may be required to locate and remove the SI and repair the surrounding damaged tissue. The MDT approach ensures a holistic, safe treatment plan, minimizing complications while restoring the patient’s function and comfort.^110,111^

## Management of the complications during retrieval or bypassing SI

Managing SI in root canals presents potential complications, particularly in narrow and curved canals.^69,70^ These risks necessitate a careful balance between the treatment success and the potential for adverse outcomes. This section provides a summary of complications that endodontists can effectively navigate challenges, minimize risks, and enhance clinical outcomes during the retrieval of SI. Below are the complications that may arise during the attempts of retrieval or bypassing SI, along with strategies to manage them:

### Tooth-related complications

- **Root perforation** Root perforation is a major complication when managing SI.^76,112^ Damage to the root canal wall integrity can severely affect the tooth’s prognosis.^112^ The risk of perforation increases when the SI is closer to the apex.^69^ Several techniques used to retrieve SI, such as modified GG drills for preparing a working platform or small files for bypassing, can lead to perforation. Perforations often occur on the inner wall of the canal curvature, similar to strip perforations. On the outer side of the curve, where bypassing efforts may cause ledging, eventually resulting in root perforation. To prevent root perforation, a careful treatment plan including preoperative radiographic evaluation to determine the instrument’s location and remaining dentin thickness is essential. The choice of bypass side should account for the root canal curvature and anatomy to minimize perforation risk. Furthermore, ultrasonic instruments should be used carefully to avoid excessive thinning of dentin. Adequate illumination, magnification, and a dry working field improve visibility and precision during retrieval.
- **Excessive removal of tooth structure** Excessive removal of tooth structure is a common complication during attempts to retrieve SI.^69,82,113^ While removing more dentin may improve the success of loosening and retrieving the fragment, it compromises the tooth’s structural integrity.^114^ Research has demonstrated that retrieving SI from the coronal one-third of the root canal does not impact the fracture resistance.^115^ In contrast, retrieval of the fragments from deeper locations within the root canal can eventually jeopardize root resistance to vertical fracture.^69,102^ Any method for removing SI should prioritize dentin preservation and minimally invasive approaches, particularly when retrieval is not necessary. To prevent this complication, endodontists should use minimally invasive techniques, high magnification, proper illumination, and employ small ultrasonic tips to vibrate around the instrument in a dry working environment to control dentin removal.
- **Thermal injury of dental and periodontal tissues** The use of ultrasonic instruments without adequate cooling can lead to excessive heat generation on the external root surface, potentially damaging periodontal ligaments and surrounding alveolar bone.^116^ In most cases, ultrasonic tips are used without coolant during the retrieval process. Studies have examined the harmful effects of increased temperature on the external root surface caused by ultrasonic when removing SI,^82,114^ indicating that the temperature increase on the external root surface is influenced by factors such as root canal wall thickness, ultrasonic tip type, power setting, and application time.^82^ Larger ultrasonic instruments cause higher temperature rise than smaller ones, although prolonged use of any size of ultrasonic tip can significantly elevate the temperature. The friction of the oscillating ultrasonic tip against the SI generates a temperature rise that is greater than that resulting from the friction against dentin.^117^ To prevent excessive temperature rise, endodontists can lower ultrasonic power settings, use smaller ultrasonic tips for precise application in an intermittent mode and irrigate frequently to dissipate heat and disinfect the root canal.

### Complications related to instrument

- **Fracture of other instruments** When attempting to bypass the fragment, a second instrument may become engaged between the SI and dentin. This scenario can cause stress exceeding the instrument’s fracture limit, leading to an additional fracture of another instrument in the root canal. To prevent this, it is essential to carefully control the power applied during instrumentation. Notably, rotating NiTi instruments are particularly unsuitable for bypassing technique due to their higher susceptibility to stress-related fracture.
- **Fracture of the original instruments** High-energy operations, such as using ultrasonic instruments, may cause the separation of the coronal portion of the original instrument. The risk depends on the material of instrument, e.g. that NiTi instruments are more prone to secondary separation than stainless steel ones. When working with NiTi instruments, ultrasonic tips should be operated at low power to reduce the risk of further separation. Additionally, techniques such as micro-tube and loop techniques can aid in safely retrieving the SI while minimizing further complications.
- **Transportation of the SI deeper into the root canal** Applying ultrasonic energy to a relatively loose separated stainless steel or NiTi instrument, especially when the ultrasonic tip is placed on the coronal end of the instrument rather than beside the fragment, may inadvertently push it deeper into the root canal. If the apical foramen is sufficiently large, the instrument may extrude through the apical constriction into periapical tissues. To prevent this, it is crucial to avoid applying apical pressure on the instrument, especially when it is located in the apical third of the root canal.
- **Dislodgement of the SI into another root canal** Once the fragment is loosened by ultrasonic energy, it may inadvertently be displaced into another root canal within the same tooth due to the flow of irrigants. In such cases, the displaced instrument can often be retrieved using irrigation, suction, or a moistened paper point. To prevent the fragment from dislodging into another root canal, it is advisable to temporarily seal other exposed root orifices in multi-canal teeth using cotton pellets, gutta-percha, or other suitable materials during retrieval.

## Future directions

Emerging technologies such as artificial intelligence (AI), bioengineering and nanotechnology are revolutionizing endodontic practice, offering novel strategies to prevent and manage instrument separation with greater accuracy and predictability.^118^

- **Artificial intelligence endodontics** AI has been applied in dental clinics, assisting endodontists by improving preoperative assessment through advanced imaging analysis, aiding in the precise localization of SI within complex root canal systems.^119,120^ AI-powered diagnostic tools integrated with CBCT can provide real-time, high-resolution visualization, facilitating accurate decision-making regarding retrieval strategies.^121^ Moreover, AI-assisted endodontics, computer-aided navigation systems and robot-assisted endodontic microsurgery may enhance precision during treatment procedures, minimizing the risk of excessive dentin removal and complications.^122^ Furthermore, machine learning models trained on large datasets can predict the risk of instrument separation based on instrument type, root canal curvature and patient-specific anatomical factors, offering potential avenues for improving diagnostic accuracy.^123^
- **Bioengineering and nanotechnology applications** Advancements in bioengineering and nanotechnology are contributing to the development of intelligent tools and therapeutic strategies for management of instrument separation. Development of smart, minimally invasive retrieval devices designed through computational modeling are enabling more conservative and efficient retrieval. Application of nanoparticle-based coatings on NiTi instruments has been shown to reduce surface friction, improve fatigue resistance, and reduce the incidence of instrument separation.^124,125^ Furthermore, nanomaterials and bioactive materials, such as lubricants, irrigants, obturating materials and sealers hold distinctive mechanical and chemical properties, enhancing post-retrieval canal disinfection and obturation.^124,126^ Recent advances in microrobotics in endodontics improves root canal disinfection and biofilm eradication in anatomically challenging regions.^127^

## Conclusion

Strict adherence to standardized protocols is indispensable in clinical practice for minimizing the risk of instrument separation. Particular caution ought to be exerted when reusing NiTi instruments. NiTi instruments are especially susceptible to fatigue and fracture in calcified or curved root canals, where increased stress is applied during instrumentation. To minimize the risk of instrument separation, instruments should be promptly replaced when encountering complex root canal anatomy or signs of wear. Considering single-use options can also further enhance the safety and efficacy of treatment.^128^ In cases of instrument separation, a thorough preoperative assessment is essential. Management strategies should be guided by a comprehensive evaluation of all relevant factors, including the characteristics of the SI, root canal anatomy, and the patient’s overall prognosis. When attempting retrieval, the likelihood of success must be carefully weighed against the risk of complications. Case selection and adherence to strict procedural protocols are critical to achieving favorable clinical outcomes. Prioritizing instrument retrieval at the expense of ignoring potential complications is strongly discouraged. The primary goal remains the successful completion of nonsurgical root canal treatment. If high-quality nonsurgical therapy fails to resolve clinical symptoms, endodontic microsurgery may be considered as an alternative to optimize outcomes and preserve the tooth.
